# Bacteriophages to Control *Campylobacter* in Commercially Farmed Broiler Chickens, in Australia

**DOI:** 10.3389/fmicb.2020.00632

**Published:** 2020-04-27

**Authors:** Helene N. Chinivasagam, Wiyada Estella, Lance Maddock, David G. Mayer, Caitlin Weyand, Phillippa L. Connerton, Ian F. Connerton

**Affiliations:** ^1^EcoSciences Precinct, Department of Agriculture and Fisheries, Queensland Government, Brisbane, QLD, Australia; ^2^Division of Microbiology, Brewing and Biotechnology, School of Biosciences, University of Nottingham, Nottingham, United Kingdom

**Keywords:** bacteriophage, *Campylobacter*, broiler chicken, Queensland (Australia), poultry

## Abstract

This study describes the development and use of bacteriophage cocktails to control *Campylobacter* in broiler chickens, in a commercial setting, in Queensland Australia, following the birds from farm to the processing plant. The components of the bacteriophage cocktails were selected to be effective against the maximum number of *Campylobacter jejuni* and *Campylobacter coli* isolates encountered on SE Queensland farms. Farms were identified that had suitable *Campylobacter* target populations and phage were undetectable 1 week prior to the intended treatment. Cocktails of phages were administered at 47 days of age. Groups of study birds were slaughtered the following day, on-farm, at the end of flock transport to the plant, and at processing (approximately 28 h post-treatment). On Farm A, the phage treatment significantly reduced *Campylobacter* levels in the ceca at the farm in the range of 1–3 log_10_ CFU/g (*p* = 0.007), compared to mock treated controls. However, individual birds sampled on farm (1/10) or following transport (2/10) exhibited high cecal *Campylobacter* counts with low phage titers, suggesting that treatment periods > 24 h may be required to ensure phage replication for effective biocontrol *in vivo*. At the time of the trial the control birds in Farm B were phage positive despite having been negative one week earlier. There was no significant difference in the cecal *Campylobacter* counts between the treatment and control groups following treatment but a fall of 1.7 log_10_ CFU/g was observed from that determined from birds collected the previous week (*p* = 0.0004). *Campylobacter* isolates from both farms retained sensitivity to the treatment phages. These trials demonstrated bacteriophages sourced from Queensland farms have the potential to reduce intestinal *Campylobacter* levels in market ready broiler chickens.

## Introduction

*Campylobacter* infection is one of the most frequently reported causes of food-borne enteritis in Australia and worldwide (Kaakoush et al., 2015). Australia began a National Notifiable Diseases Surveillance System in 1991 and since then, the number of cases of human infection has steadily risen to 137.5/100,000, in 2018^1^. This incidence rate is higher than many other parts of the world (Kaakoush et al., 2015) and it is estimated that only around 10% of *Campylobacter* infection cases are recorded (Hall et al., 2008). Thus, *Campylobacter* has a significant impact on the health and economic prosperity of the Australia population.

Consumption of poultry meat has been identified as an important risk factor for human infection from source attribution studies (Mughini Gras et al., 2012; Ravel et al., 2017). *Campylobacter* spp., particularly *Campylobacter jejuni* and to a lesser extent *Campylobacter coli* are ubiquitous in the intestinal contents of broiler chickens (European Food Safety Authority [EFSA] and European Centre for Disease Prevention and Control [ECDC], 2018). During slaughter and processing, poultry carcasses frequently become contaminated with *Campylobacter* from the intestinal contents, providing a reservoir for human infection (European Food Safety Authority [EFSA], 2010). Consequently, managing *Campylobacter* numbers in poultry hosts, on-farm, is a promising strategy to reduce disease burden. A 3 log_10_ reduction in *Campylobacter* numbers in the intestines of infected birds at slaughter, could potentially contribute to a 90% reduction in public health risks (Crotta et al., 2017). European studies indicate that on-farm interventions can be very effective with a 1.0 log_10_ reduction in fecal count supported by a 1.0 log_10_ reduction in contamination of the exterior of chickens (during processing) could result in a 90% reduction of human infections (Havelaar et al., 2007). Biocontrol using bacteriophages has the potential to control *Campylobacter* numbers in highly contaminated flocks (Crotta et al., 2017).

Biocontrol using bacteriophages has been exploited for controlling other food-borne pathogens such as *Salmonella*, *Escherichia coli* O157:H7 and *Listeria monocytogenes* (Goodridge and Bisha, 2011). Bacteriophages have been shown to be naturally present in poultry environments in the United Kingdom and Australia along with their host campylobacters (Connerton et al., 2004; Loc Carrillo et al., 2007; Owens et al., 2013). Poultry farms are a natural source of phages from which to develop appropriate on-farm treatments as their use will not introduce any agent that is not already frequently encountered (Atterbury et al., 2005; El-Shibiny et al., 2005). These considerations are of commercial importance against a background of consumer anxiety regarding the adoption of intervention strategies against campylobacters from poultry (MacRitchie et al., 2014). Experimental studies have demonstrated the potential to use phages to reduce *C. jejuni* in broiler chicken ceca (Loc Carrillo et al., 2005; El-Shibiny et al., 2009) and the surface of chicken skin (Atterbury et al., 2003; Goode et al., 2003). Bacteriophages can be used to reduce *Campylobacter* either on-farm or on the processed product (Connerton et al., 2011). A one log_10_ reduction in the numbers of *C. jejuni* and *C. coli* in feces has been reported (Carvalho et al., 2010). The use of phages on-farm is a welfare friendly option for the biological control of *Campylobacter* that can be adopted from a logistic perspective for use with commercial poultry.

Phage selection, method, and timing of application on commercial farms are important criteria to achieve meaningful reductions in terms of the risk to the consumer. Wagenaar et al. (2005) have demonstrated 1–3 log_10_ reductions in *Campylobacter* counts at various points in the rearing cycle. Loc Carrillo et al. (2005) have also demonstrated log reductions (0.5 and 5 log_10_ CFU/g) over an extended treatment period (5 days) on experimentally infected birds (25 days old). Similarly, *Campylobacter* reductions in ceca have been demonstrated over a shorter period (i.e., 2 log_10_ CFU/g reduction over 48 h) in experimentally inoculated broilers (El-Shibiny et al., 2009). Treatment of naturally colonized commercial birds has been demonstrated to produce significant reductions in *Campylobacter* levels of 3.2 log_10_ CFU/g of ceca from one of the three commercial broiler houses involved in the trial (Kittler et al., 2013).

The present study was designed to provide a better understanding of the application of phages for typical Australian commercial farm settings. *Campylobacter* counts were assessed at the end of rearing on the farm, after transport, and on carcasses post processing.

## Methodology

### *Campylobacter* Strains

*Campylobacter* strains used as bacteriophage hosts were as follows: *C. jejuni* NCTC 12662, *C. jejuni* NC3142 (Farm C isolate sourced from re-use litter in 2011), and *C. coli* NC2934 (Farm C isolate sourced from non-reused litter in 2011). These were grown on Blood agar No 2 (Oxoid CM0271) with 5% (v/v) lyzed horse blood added (LHB; Oxoid Australia), for 24 h at 42°C, under microaerobic conditions, produced by using Campygen gas packs (Oxoid, CN0025A; Basingstoke, United Kingdom). Isolates used for resistance testing were sourced from chicken ceca and carcasses pre- and post-initiation of the study.

### Bacteriophage Isolation, Characterization, and Selection

Bacteriophages used in this study were isolated between 2012 and 2015, from samples of: cecal contents, litter, carcass rinses, and soil from Queensland broiler chicken farms and pig effluent. For direct isolation from cecal contents, a 10% suspension in SM buffer (100 mM NaCl; 8 mM MgSO_4_.7H_2_O; 0.01% gelatin; 50 mM Tris-HCl, pH7.5), incubated over-night at 4°C, centrifuged at 15,000 *g* for 10 min at 4°C, filtered through 0.2 μm filters (Minisart; Sartorius) then 100 μl added containing 200 μl of *Campylobacter* host strains (10^8^ CFU/ml) and incubated at 42°C for 30 min to allow phage to bind to host. The proportion of sample to broth was as follows: cecal contents and pig effluent 1:4; litter 1:6; soil 1:3, and carcass rinse 1:2. The suspension was the added to 5 ml of molten 0.6% NZCYM (Difco, Beckton Dickinson, United States) soft agar overlay at 48°C, poured onto 1% NZCYM plates and allowed to set. The plates were then dried with lids partially open and then incubated at 42°C for 24 h, under microaerobic conditions (Connerton et al., 2004). Isolated plaques were collected using a pipette tip and suspended in 100 μl of SM buffer. Each single plaque was propagated three times to ensure that the isolates represented a single clone. In addition, an enrichment technique using modified Preston broth (Bolton et al., 1983) was used. The base Preston broth was prepared from Nutrient Broth Number 2 (NB2; Oxoid CM0067), 5% (v/v) LHB (Oxoid), with *Campylobacter* growth supplement (Oxoid, SR0232) and Preston *Campylobacter* selective supplement (Oxoid SR0117). This was modified for phage enrichment by addition of 10 mM MgSO_4_7H_2_O and 1 mM CaCl_2_ to stabilize phage capsids (Adams, 1959). Fifty microliters of overnight cultures of *C. jejuni* 12662, *C. jejuni* NC3142, and *C. coli* NC2934 (that had been grown in NB2 with 5% LHB (v/v) and incubated overnight at 42°C) were added to the diluted samples. The enrichment broth containing sample and host bacteria were incubated at 42°C for 24 h under microaerobic conditions. After incubation, centrifugation, and filtration, phages were isolated using the soft agar technique as described above for direct isolation, using all three hosts on separate plates. Approximately 600 bacteriophages were isolated using these methods from using either direct isolation or enrichment. A total of 128 (from the 600) phages were screened (described below) against 486 *Campylobacter* isolates using multiple combinations to narrow down representation to a 17-member phage panel. A further two phages from Queensland pig farm effluent were also included based on their lytic activity to arrive at a 19-member cocktail candidate panel. The *Campylobacter* isolates used, represented a mix of *C. jejuni* and *C. coli*, sourced from 17 South East Queensland farms across 36 farm samplings that occurred from 2009 to 2013.

### Screening of Farm *Campylobacter* Isolates Against Phage Cocktail Candidates

The lytic activities of 19 phage cocktail candidates were tested against 241 representative *Campylobacter* isolates sourced between 2012 and 2016 from nine different Queensland farms. The 19 phage cocktail candidates, diluted to contain 10^6^ PFU/ml were dispensed as 10 μl droplets, onto test bacterial lawns prepared as above. Following incubation, strains were scored as sensitive to a bacteriophage, if clear lysis or semi-clear lysis was observed following incubation.

### Pre-screening of Birds to Enable Farm Selection

Six farms all having birds of roughly the same age were pre-screened approximately 1 week before the end of the growth cycle. Three chickens were randomly picked from two to four sheds. The criteria for final selection was that the farm should have a high-level resident *Campylobacter* population that was sensitive to more than one of the 19 selected phage candidates, and that the digesta samples were negative for indigenous phage. The absence of indigenous phage was considered a prerequisite because a previous survey demonstrated that such phage can effect mean *Campylobacter* populations (Atterbury et al., 2005). Birds were euthanized at the farm and the ceca were removed before transport on ice to the laboratory within 3–4 h. For *Campylobacter* enumeration, serial dilutions of cecal contents were prepared in MRD then 100 μl of each dilution was spread in triplicate, onto mCCDA (Oxoid CM0739) plates, containing selective supplement (Oxoid SR0155), and then incubated at 37°C for 48 h under microaerobic conditions as above. Ten well-separated *Campylobacter* colonies per shed were randomly picked across the three samples and re-streaked for purity. Their lysis profiles were then assessed against the 19 phage cocktail candidates (described above). Two farms which fulfilled the three criteria were selected (Farm A and Farm B) and the most appropriate phages selected to form a cocktail to administer to the test birds on these farms. These were PH5, PH8, PH11, and PH13 for Farm A and PH18 and PH19 for Farm B.

### Farm Trials

Descriptions of the two test farms and conditions employed during the trial are presented in Table 1. For each of the two selected farms, two groups of randomly picked 30 birds (phage treated and placebo groups) were segregated into two pens, within the chicken barn using wire mesh, but all other farming conditions remained same as the rest of barn. All the birds were 47 days of age at this point. Phage or placebo were administered by oral gavage, 1 day prior to transport to the processing plant. The placebo group of chickens received 3 ml of sterile tap water while the phage treatment group were given 3 ml of 10^7^ PFU/ml of each phage combined in sterile tap water. Food and water were withdrawn 8 h prior to collection according to normal farm practice. The following day, when the main cohort were collected for transport to the processing plant, 10 birds from each group were euthanized at the farm. A further 10 birds were euthanized following transport (which took approximately 4 h) and the carcasses of the final group were processed by the plant and collected before the chlorine rinse stage.

**TABLE 1:** Description of trial farms and conditions relevant to trial.

**Farm situation**	**Farm A**	**Farm B**
Shed dimensions	153 × 15.2 m	122 × 13.7 m
Shed area	2325 m^2^	1670 m^2^
Pen sizes	6 m (L) × 1.5 (W) = 9 m^2^	4 m (L) × 2.5 (W) = 10 m^2^
Distance between treatment and control	5.5 m	4.5 m
Birds remaining at dosing	16,800	12,750
Bird density	7.22/m^2^	7.63/m^2^
Bird age at pick-up	48 days	48 days
Litter practice	Australian partial re-use	Australian partial re-use

**Other commercial practices relevant to the trial birds**	

Feed withdrawal	8 h prior pick-up (as others)
Water withdrawal	1 h prior pick-up (as others)
Transport	Placed in designated transport module and transport in truck with other commercial birds
Processing	Moved along process chain along with other non-trial birds and removed prior chlorination

### Sample Preparation and Enumeration of *Campylobacter*

Chicken ceca were removed aseptically and transported to the laboratory on ice. The ceca were chopped into fine material, from which 10 g samples were aseptically blended using a homogenizer for 1 min, with a diluent consisting of NB2 with LHB 5% (v/v). A 10 cm length of the ileum was measured from the ileal–cecal junction similarly transported on ice to laboratory. The total ileum contents were weighed, and appropriate 10-fold dilutions prepared. Carcasses were removed prior to chlorination and spin-chilling and placed in a sterile bag with 200 ml of 0.1% peptone (Oxoid LP0037) and shaken for 2 min in a shaker designed for the purpose. Ten-fold dilutions were prepared using Preston broth, and *Campylobacter* enumerated using a three tube, MPN technique for Farm B (Chinivasagam et al., 2009). Briefly the dilution tubes were incubated at 42°C for 48 h under microaerobic conditions, then sub-cultured on mCCDA and further incubated at 42°C for 48 h under microaerobic conditions, and finally scored as positive or negative. The MPN was calculated using tables with correction for dilution.

### Enumeration of Phage in Ceca, Ileum and on Carcasses

Bacteriophages were enumerated from cecal and ileum contents by decimal dilution in SM buffer as described above. For carcass rinses, the suspension used for *Campylobacter* enumeration was diluted 1:1 in SM buffer and then treated as for the ceca and ileum samples.

### Statistical Analysis

Data were analyzed using Prism8 (GraphPad Software, San Diego, CA, United States). The Shapiro–Wilks test of normality was employed on all log_10_-transformed bacterial counts or phage titers. Non-parametric tests were used as indicated when the data did not conform to normality at a significance level of 0.05. Spearman’s rank correlation coefficients were calculated using Prism8 to assess rank correlations between two variables from non-parametric data.

## Results

### Diversity of the 19-Phage Panel Against a Selection of Farm *Campylobacter* Isolates

The selected cocktail candidates demonstrated diversity in their lytic activity against farm *Campylobacter* isolates. Among the 241 farm *Campylobacter* isolates compared, 200 were identified as *C. jejuni*, 39 were *C. coli*, and two were not speciated. One hundred and eighty-eight of the *Campylobacter* strains (78%) were sensitive to at least one member of the bacteriophage panel. The results for each bacteriophage are shown in Table 2.

**TABLE 2:** The panel of 19 bacteriophages were tested for sensitivity to 241 *Campylobacter* hosts.

**Phage**	**Number of sensitive *Campylobacter* hosts**	**Sensitive *C. jejuni* (% of total number tested)**	**Sensitive *C. coli* (% of total number tested)**
PH 1	53	26	0
PH 2	65	30	13
PH 3	60	27	13
PH 4	55	27	3
PH 5	89	43	5
PH 6	77	35	18
PH 7	28	14	0
PH 8	83	38	15
PH 9	66	30	13
PH 10	69	34	0
PH 11	72	34	8
PH 12	64	29	13
PH 13	37	18	3
PH 14	31	15	3
PH 15	46	22	3
PH 16	76	36	8
PH 17	54	27	0
PH 18	128	48	82
PH 19	153	61	79

This analysis revealed that bacteriophages PH18 and PH19 had the broadest lytic activity, and although effective against most *C. jejuni* strains appeared to be more efficacious against *C. coli*. In contrast, some bacteriophages, for example, bacteriophages PH1, PH7, PH19, and PH17, were active against multiple *C. jejuni* strains, but did not lyze any of the *C. coli* isolates tested.

### Selection of Suitable Locations for On-Farm Treatment Trials

Table 3 presents the *Campylobacter* and phage counts and *Campylobacter* species identity for 18 sheds screened across six potential farms. The lytic profiles for each of the 10 isolates tested were similar indicating that a single *Campylobacter* type was dominant at that point in the rearing cycle. All isolates tested from these six farms represented both *C. jejuni* and *C. coli*, with *C. jejuni* being dominant (Table 3) Rejected farms Farm D and F had a phage presence (though those farms met the other phage candidate selection criteria). The criteria for *Campylobacter* were met across all six farms which ranged from log_10_ 7.00 to 9.00 CFU/g. For Farm A, all 10 isolates were sensitive to 13 out of the 19 candidate phages with only one phage having no activity (presented in Supplementary Table S1). Phages PH5, PH8, PH11, and PH13 were selected from the 13 candidates for the Farm A trial. For Farm B, Phages PH1–PH17 showed no activity against any of the 10 test isolates, but all the isolates were sensitive to PH18 and PH19 (Supplementary Table S1), which were therefore selected for Farm B trial.

**TABLE 3:** Pre-screening the cecal contents from 40 days birds to select test farms.

**Farm**	**Shed**	***Campylobacter* log_10_ CFU/g^1^**	**Species**	**Phage log_10_ PFU/g^1^**
A	1	8.56	all *C. jejuni*	<2
	2	8.35	all *C. jejuni*	<2
	**7**	**8.59**^ < *cps*⁣:*bf*⁣ > 2⁣ < ⁣/*cps*⁣:*bf*⁣ >^	all *C. jejuni*	**<2**
B	1	7.00	all *C. jejuni*	<2
	2	6.97	all *C. jejuni*	<2
	**3**	**7.84**^ < *cps*⁣:*bf*⁣ > 2⁣ < ⁣/*cps*⁣:*bf*⁣ >^	all *C. jejuni*	**<2**
	B	7.2	all *C. jejuni*	<2
C	7	7.53	all *C. jejuni*	<2
	8	7.89	all *C. jejuni*	<2
D	1	7.12	all *C. jejuni*	5.30
	3	8.16	all *C. jejuni*	5.70
	5	7.37	all *C. jejuni*	ND^3^
E	1	8.98	all *C. jejuni*	<2
	2	8.78	all *C. jejuni*	<2
	3	8.7	all *C. jejuni*	<2
F	1	7.00	all *C. coli*	<2
	2	9.08	all *C. coli*	<2
	3	9.08	all *C. coli*	3.66

*^1^Value represents the mean *Campylobacter* count or mean phage titer from the cecal contents of three randomly sampled birds, where the limit of detection was 2 log_10_ PFU/g1. ^2^Farm/shed selected for trial. ^3^ND, not determined.*

### Enumeration of *Campylobacter* and Phages From Intestinal Contents of Birds On-Farm and at the Plant

**FIGURE 1:**
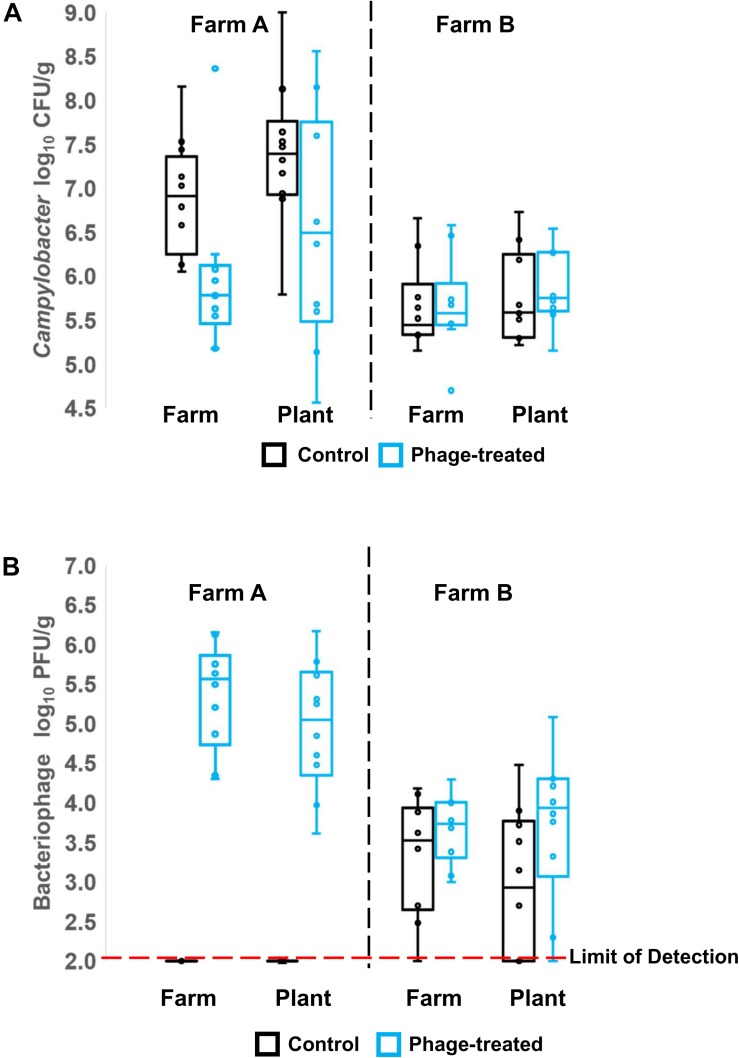
Box Whisper plots showing: **(A)** enumeration of *Campylobacter* in cecal contents, in control and phage-treated birds, comparing values on the farm and at the plant (after transport); **(B)** enumeration of bacteriophage in cecal contents, in control and phage-treated birds, comparing values on the farm and at the plant (after transport).

*Campylobacter* and phages were enumerated from cecal and ileal samples of the birds before transport and after transport, but only phages were enumerated from the processed carcasses of Farm B, due to high levels of competitive flora overgrowing the *Campylobacter* selective medium. Unlike the control log_10_ CFU/g *Campylobacter* counts from the ceca, the counts for the phage-treated birds were not normally distributed from Farm A (Shapiro–Wilk test). The median for the control was 6.91 log_10_ CFU/g compared to 5.79 log_10_ CFU/g for the phage treated birds (Figure 1A, Farm A). Non-parametric analysis indicates the phage treated cecal *Campylobacter* counts were significantly lower than controls (*p* = 0.007; Mann–Whitney *U*-test). The data feature an outlier of 8.36 log_10_ CFU/g that was investigated by reference to repeat enumeration data at 24 h and the phage titer recorded for the bird. The cecal *Campylobacter* count was confirmed and included in the analysis, but it was noted that the phage titer for the sample was the lowest recorded and may represent a situation where the phages have not efficiently replicated throughout the digesta in the treatment period. Following transport to the plant, no significant difference (*p* = 0.242; Mann–Whitney *U*-test) was observed between the *Campylobacter* counts in phage-treated and control birds (Figure 1A; Farm A), although the median for the control was greater at 7.39 log_10_ CFU/g compared to 6.49 log_10_ CFU/g for the phage treated birds. The *Campylobacter* counts for the phage-treated data also contained two high cecal counts > 8.0 log_10_ CFU/g that correspond with low phage titers. Analysis of the data using the Spearman’s rank correlation coefficient indicates a strong negative correlation is evident between the *Campylobacter* count and the phage titer in the treatment birds (r_s_ = −0.746; *p* = 0.02). Figure 1B shows the titers of bacteriophages recovered on-farm and at the plant for Farm A (left panel). As expected, for the control birds from Farm A, bacteriophages were not detected (below limit of detection). There was no significant difference between farm and plant for those birds that had been treated with bacteriophage with median values of 5.6 and 5.0 log_10_ PFU/g cecal contents, respectively.

Figure 1A (right panel) shows the *Campylobacter* enumeration data for the cecal contents of birds from Farm B, on-farm and after transport to the plant. *Campylobacter* counts in the control and phage-treated birds either on farm (*p* = 0.373; Mann–Whitney *U*-test) or after transport (*p* = 0.384; Mann–Whitney *U*-test) were not significantly different. Enumeration of *Campylobacter* from the ileum did not show any significant difference at either farm or plant (data not shown). However, the phage titer data for farm B were unexpected in that control birds were colonized by phage despite having been administered a placebo and had been selected as phage-negative at pre-screen 1 week earlier (Figure 1B, right panel). The difference in the mean *Campylobacter* counts from Farm B at pre-screen and the experimental values of the control birds recorded on-farm represents a significant reduction of 1.7 log_10_ CFU/g (*p* = 0.006; Mann–Whitney *U*-test).

### Enumeration of *Campylobacter* on Carcasses

The *Campylobacter* numbers recovered from carcasses comparing treated and control birds from Farm B were not significantly different (*p* = 0.406; Mann–Whitney *U*-test), although six carcasses were below detection limit of <6000 organisms per carcass. Bacteriophage were detected at low titer (<100 PFU/carcass) from four carcasses of Farm B, but not detected on any carcasses from Farm A.

### Continued Sensitivity to Isolates to Cocktail Before and After Phage-Treatment

The lytic profile of the cocktail phages to *Campylobacter* strains isolated after phage treatment, from both farms were unchanged compared to those isolated before treatment (Table 4). Development of resistance to the treatment phages was therefore not detected.

**TABLE 4:** Lytic profiles of *Campylobacter* isolates from Farm A and Farm B, to the candidate bacteriophage pre-and post-treatment, and after transport to the plant.

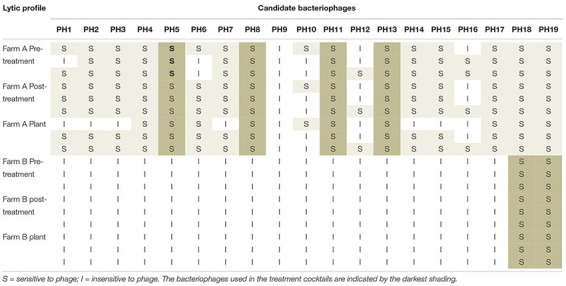

*S = sensitive to phage; I = insensitive to phage. The bacteriophages used in the treatment cocktails are indicated by the darkest shading.*

## Discussion

One of the reasons that phage therapy was superseded by antibiotic therapies is that utilizing a biological agent requires care in selection of the appropriate agent and in application. However, the subtlety of being able to target pathogenic species within a complex microbiota, without causing dysbiosis represents a major advantage. Campylobacters are not overt pathogens of chickens so the ability to target the zoonotic component of the microbiota is a key advantage to the welfare of the bird and the quality of the product (Richards et al., 2019). However, for success the bacteriophages must be virulent against the target bacteria, and able to reach the target in sufficient quantities to affect a change in the host bacterial population. Exposure time is also a key consideration for a self-amplifying antimicrobial. Phages must have enough time to achieve a titer that will enable their access to all host-rich environments within the gut to kill the target bacteria. Intestinal transit will limit the exposure time to high titers, which will limit the development of resistance in the target populations. Experiments in the laboratory have gone a long way to understanding what is required for successful reduction of *Campylobacter* in chickens but studies are required to assess operation in commercial settings where the birds are subjected to feed withdrawal and the stress of transportation.

Selecting of the optimum phages from a panel that are active against this dominant *Campylobacter* is key to a successful intervention. Data from *Campylobacter* and non-*Campylobacter* studies suggest that the optimal number of phages in a cocktail is between two and four (for examples, see Carvalho et al., 2010; Fischer et al., 2013; Manohar et al., 2019). For Farm A, where 17 phages were active against the host bacteria, using four as the maximum was a logical choice. However, for Farm B, only two of the candidate phages had activity to the *Campylobacter* host, so only two phages were used in the cocktail. The use of the four-phage cocktail on Farm A brought about a significant decline in the *Campylobacter* count compared to control birds within the ceca that represents the major reservoir of intestinal contamination.

Once the birds had been transported to the processing plant, it is possible that the increases in the mean *Campylobacter* counts for both phage-treated and control birds were due to transport stress. Increases in *Campylobacter* count following transport have been noted previously for cecal content and fecal matter, which have been postulated to arise as a result of the effects of transport on peristaltic movements of the chicken gut (Stern et al., 1995; Whyte et al., 2001). This was not manifest as a consistent increase in all birds, but it appeared that some birds were more affected than others, illustrated by the wide range of counts in the phage-treated birds including some that were very high at approximately 8 log_10_ CFU/g and some that were below 6 log_10_ CFU/g. The data do, however, demonstrate a strong negative correlation between the *Campylobacter* count and the phage titer. The high *Campylobacter* counts in some of the phage-treated birds may therefore represent birds where the phages have not attained a great enough titer in the ceca to affect a reduction in the population. All birds were treated similarly so the failure is likely due to low host concentrations encountered in the amplification phase, for example, in the intestinal tract prior to reaching the ceca. Success under these circumstances would then be reliant on titer amplification and dissemination in the ceca, which is subject to regular cecal evacuation. The treatment period to affect the *Campylobacter* reductions in as many birds as possible needs to be increased from 24 h as the phage may not have sufficient time to achieve the titer and/or the required dispersion within the intestine. However, in the majority of the phage treated birds of Farm A, the phage did replicate and were effective at reducing *Campylobacter* numbers in the cecal contents.

The main difference from experimental models is that birds become naturally infected with *Campylobacter* strains that provoke competition with each other. Although dominant types can emerge that predominate in surveys (El-Shibiny et al., 2005). Similarly, biosecurity measures applied on a commercial farm may not prevent the incursion of native phages from the environment. Phage isolation studies report variable frequencies of between 20 and 50% recovery from chicken sources, which suggests phages are not always present in *Campylobacter* infected flocks (Atterbury et al., 2005; El-Shibiny et al., 2005; Owens et al., 2013). Campylobacters exposed to phage can become resistant to the infecting phage; however, there is frequently an associated cost in competitive fitness (Connerton et al., 2004; Scott et al., 2007). Thus, selection favors phage-sensitive hosts when phages are not present or are at low titers. Commercial farms are challenged by multiple campylobacters that undergo succession, and one facet of the competition is the evasion of phage. A longitudinal study across successive flocks of a *Campylobacter* and phage infected barn resulted in the elimination of the phage when a new phage insensitive strain entered the environment to dominate (Connerton et al., 2004). This scenario is consistent with evidence from Farm B that became colonized by a previously undetectable phage that proliferated on sensitive *Campylobacter* populations in the final week of rearing after the pre-screen. However, we also acknowledge that the presence of an indigenous phage, albeit not detected at pre-screen, may also have affected the narrow choice of treatment phage able to lyse campylobacters from Farm B. A similar situation was also reported by Kittler et al. (2013), in one of three sites investigated. The infiltrating phages clearly spread rapidly throughout the flock and it seems likely that they reduced the numbers of *Campylobacter* in cecal contents of both control and test birds, either before or concurrently, with the phage intervention. At the time of sampling, the average *Campylobacter* number in the cecal contents was 5.6 log_10_ CFU/g in contrast to 7.8 log_10_ CFU/g, when pre-screened, 1 week earlier, with no phages detected. While this makes the results difficult to interpret with respect to the treatment phage, it sheds light on the process of concurrent phage infection that are part of the natural *Campylobacter*–phage interactions occurring in the intestinal tracts of farm chickens, and confirms earlier reports that the presence of phage in broiler flocks can reduce cecal *Campylobacter* population levels (Atterbury et al., 2005). Reduced concentrations of the target bacteria below the phage proliferation threshold will also diminish the likelihood of *in situ* amplification of the treatment phage and the effectiveness of the intervention (Cairns et al., 2009). These observations suggest that the selection of the treatment phage should also take in to account the action of concurrent phage infections, which may make phage intervention more effective if optimized correctly.

The development of resistance to phage treatment is often cited as a negative aspect of phage intervention but resistance to the phage cocktails selected was not detected on either farm. This may have been partly because the time between intervention and slaughter was less than 24 h, which reduces the time for resistant strains to emerge although this was also minimized by using cocktails of phages rather than just one. Increasing the time between treatment and slaughter from 24 to 2–4 days may increase the effectiveness of treatment (Loc Carrillo et al., 2005; Wagenaar et al., 2005; El-Shibiny et al., 2009; Kittler et al., 2013) but may also lead to the emergence of resistance. The short exposure of the birds to the phage cocktail treatment may have contributed to low phage titers observed and the strong correlation with the *Campylobacter* counts observed on Farm A in the absence of emerging phage resistant populations. Phage resistant mutants were also absent amongst the campylobacters recovered from birds of Farm B that had been pre-exposed to indigenous phage. A combination of competition and exposure to new virulent phage may have eliminated any resistant types in the treatment group. However, phage insensitive isolates were also not evident from the control group *Campylobacter* isolates. The residual population surviving intestinal colonization by the indigenous phage may not have been exposed to the phage to become resistant, and similarly were either not exposed to the treatment phage or not exposed to sufficient phage titer to affect a further reduction in the colonization level. This study highlights a general need to understand system specific phage–host interactions that are likely critical for successful treatment outcomes.

## Data Availability Statement

The datasets generated for this study are available on request to the corresponding author.

## Ethics Statement

The experimental design was approved by the Animal Ethics Committee of the Department of Agriculture and Fisheries Queensland before commencing farm screening and experimental intervention trials. Broiler chickens were reared in accordance with the National Animal Welfare Standards for the Chicken Meat Industry (available at: chicken.org.au/wp-content/uploads/2017/09/ME-083-Chicken-Standards-The-Standards-3.pdf) and the National Farm Biosecurity Manual for chicken growers (available at: chicken.org.au/wp-content/uploads/2017/09/National-Farm-Biosecurity-Manual-for-Chicken-Growers-Feb-2010-web.pdf).

## Author Contributions

HC and IC designed the experiments. LM executed the farm trials, WE, LM, CW, and HC executed the lab experiments. HC, PC, IC, and DM analyzed the data. HC, PC, and IC prepared the manuscript.

## Conflict of Interest

The authors declare that the research was conducted in the absence of any commercial or financial relationships that could be construed as a potential conflict of interest.

## Footnotes

1 http://www9.health.gov.au/cda/source/rpt_4.cfm

## References

[B1] Adams M. H. (1959). *Bacteriophages.* New York, NY: Interscience Publishers Inc.

[B2] Atterbury R. J. Connerton P. L. Dodd C. E. Rees C. E. Connerton I. F. (2003). Application of host-specific bacteriophages to the surface of chicken skin leads to a reduction in recovery of *Campylobacter jejuni*. *Appl. Environ. Microbiol.* 69 6302–6306. 10.1128/aem.69.10.6302-6306.2003 14532096 PMC201188

[B3] Atterbury R. J. Dillon E. Swift C. Connerton P. L. Frost J. A. Dodd C. E. (2005). Correlation of *Campylobacter* bacteriophage with reduced presence of hosts in broiler chicken ceca. *Appl. Environ. Microbiol.* 71 4885–4887. 10.1128/aem.71.8.4885-4887.2005 16085889 PMC1183290

[B4] Bolton F. J. Coates D. Hinchliffe P. M. Robertson L. (1983). Comparison of selective media for isolation of *Campylobacter jejuni/coli*. *J. Clin. Pathol.* 36 78–83. 10.1136/jcp.36.1.78 6822680 PMC498109

[B5] Cairns B. J. Timms A. R. Jansen V. A. Connerton I. F. Payne R. J. (2009). Quantitative models of *in vitro* bacteriophage-host dynamics and their application to phage therapy. *PLoS Pathog.* 5:e1000253. 10.1371/journal.ppat.1000253 19119417 PMC2603284

[B6] Carvalho C. Susano M. Fernandes E. Santos S. Gannon B. Nicolau A. (2010). Method for bacteriophage isolation against target *Campylobacter* strains. *Lett. Appl. Microbiol.* 50 192–197. 20002571 10.1111/j.1472-765X.2009.02774.x

[B7] Chinivasagam H. N. Tran T. Maddock L. Gale A. Blackall P. J. (2009). Mechanically ventilated broiler sheds: a possible source of aerosolized *Salmonella*, *Campylobacter*, and *Escherichia coli*. *Appl. Environ. Microbiol.* 75 7417–7425. 10.1128/aem.01380-09 19801461 PMC2786424

[B8] Connerton P. L. Loc Carrillo C. M. Swift C. Dillon E. Scott A. Rees C. E. (2004). Longitudinal study of *Campylobacter jejuni* bacteriophages and their hosts from broiler chickens. *Appl. Environ. Microbiol.* 70 3877–3883. 10.1128/aem.70.7.3877-3883.2004 15240258 PMC444807

[B9] Connerton P. L. Timms A. R. Connerton I. F. (2011). *Campylobacter* bacteriophages and bacteriophage therapy. *J. Appl. Microbiol.* 111 255–265. 10.1111/j.1365-2672.2011.05012.x 21447013

[B10] Crotta M. Georgiev M. Guitian J. (2017). Quantitative risk assessment of *Campylobacter* in broiler chickens – Assessing interventions to reduce the level of contamination at the end of the rearing period. *Food Control* 75 29–39. 10.1016/j.foodcont.2016.12.024

[B11] El-Shibiny A. Connerton P. L. Connerton I. F. (2005). Enumeration and diversity of campylobacters and bacteriophages isolated during the rearing cycles of free-range and organic chickens. *Appl. Environ. Microbiol.* 71 1259–1266. 10.1128/aem.71.3.1259-1266.2005 15746327 PMC1065130

[B12] El-Shibiny A. Scott A. Timms A. Metawea Y. Connerton P. Connerton I. (2009). Application of a group II Campylobacter bacteriophage to reduce strains of *Campylobacter jejuni* and *Campylobacter coli* colonizing broiler chickens. *J. Food Prot.* 72 733–740. 10.4315/0362-028x-72.4.733 19435220

[B13] European Food Safety Authority [EFSA] (2010). Scientific Opinion on Quantification of the risk posed by broiler meat to human campylobacteriosis in the EU. *EFSA J.* 8:1437 10.2903/j.efsa.2010.1437 PMC13129989 42077633

[B14] European Food Safety Authority [EFSA], and European Centre for Disease Prevention and Control [ECDC] (2018). The European Union summary report on trends and sources of zoonoses, zoonotic agents and food-borne outbreaks in 2017. *EFSA J.* 16:5500 10.2903/j.efsa.2018.5500 PMC7009540 32625785

[B15] Fischer S. Kittler S. Klein G. Glünder G. (2013). Impact of a single phage and a phage cocktail application in broilers on reduction of *Campylobacter jejuni* and development of resistance. *PLoS One* 8:e78543. 10.1371/journal.pone.0078543 24205254 PMC3804501

[B16] Goode D. Allen V. M. Barrow P. A. (2003). Reduction of experimental *Salmonella* and *Campylobacter* contamination of chicken skin by application of lytic bacteriophages. *Appl. Environ. Microbiol.* 69 5032–5036. 10.1128/aem.69.8.5032-5036.2003 12902308 PMC169133

[B17] Goodridge L. D. Bisha B. (2011). Phage-based biocontrol strategies to reduce foodborne pathogens in foods. *Bacteriophage* 1 130–137. 10.4161/bact.1.3.17629 22164346 PMC3225777

[B18] Hall G. Yohannes K. Raupach J. Becker N. Kirk M. (2008). Estimating community incidence of *Salmonella*, *Campylobacter*, and Shiga toxin-producing *Escherichia coli* infections, Australia. *Emerg. Infect. Dis.* 14 1601–1609. 18826825 10.3201/eid1410.071042 PMC2609882

[B19] Havelaar A. H. Mangen M. J. de Koeijer A. A. Bogaardt M. J. Evers E. G. Jacobs-Reitsma W. F. (2007). Effectiveness and efficiency of controlling *Campylobacter* on broiler chicken meat. *Risk Anal.* 27 831–844. 10.1111/j.1539-6924.2007.00926.x 17958495

[B20] Kaakoush N. O. Castaño-Rodríguez N. Mitchell H. M. Man S. M. (2015). Global epidemiology of *Campylobacter* infection. *Clin. Microbiol. Rev.* 28 687–720. 10.1128/cmr.00006-15 26062576 PMC4462680

[B21] Kittler S. Fischer S. Abdulmawjood A. Glünder G. Klein G. (2013). Effect of bacteriophage application on *Campylobacter jejuni* loads in commercial broiler flocks. *Appl. Environ. Microbiol.* 79 7525–7533. 10.1128/aem.02703-13 24077703 PMC3837725

[B22] Loc Carrillo C. Atterbury R. J. El-Shibiny A. Connerton P. L. Dillon E. Scott A. (2005). Bacteriophage therapy to reduce *Campylobacter jejuni* colonization of broiler chickens. *Appl. Environ. Microbiol.* 71 6554–6563. 10.1128/aem.71.11.6554-6563.2005 16269681 PMC1287621

[B23] Loc Carrillo C. M. Connerton P. L. Pearson T. Connerton I. F. (2007). Free-range layer chickens as a source of *Campylobacter* bacteriophage. *Antonie Van Leeuwenhoek* 92 275–284. 10.1007/s10482-007-9156-4 17387630

[B24] MacRitchie L. A. Hunter C. J. Strachan N. J. C. (2014). Consumer acceptability of interventions to reduce *Campylobacter* in the poultry food chain. *Food Control* 35 260–266. 10.1016/j.foodcont.2013.06.005 24882947 PMC4029083

[B25] Manohar P. Tamhankar A. J. Lundborg C. S. Nachimuthu R. (2019). Therapeutic characterization and efficacy of bacteriophage cocktails infecting *Escherichia coli*, *Klebsiella pneumoniae*, and *Enterobacter* species. *Front. Microbiol.* 10:574 10.3389/fmicb.2019.00574 PMC6437105 30949158

[B26] Mughini Gras L. Smid J. H. Wagenaar J. A. de Boer A. G. Havelaar A. H. Friesema I. H. (2012). Risk factors for campylobacteriosis of chicken, ruminant, and environmental origin: a combined case-control and source attribution analysis. *PLoS One* 7:e42599. 10.1371/journal.pone.0042599 22880049 PMC3411806

[B27] Owens J. Barton M. D. Heuzenroeder M. W. (2013). The isolation and characterization of *Campylobacter jejuni* bacteriophages from free range and indoor poultry. *Vet. Microbiol.* 162 144–150. 10.1016/j.vetmic.2012.08.017 22980913

[B28] Ravel A. Hurst M. Petrica N. David J. Mutschall S. K. Pintar K. (2017). Source attribution of human campylobacteriosis at the point of exposure by combining comparative exposure assessment and subtype comparison based on comparative genomic fingerprinting. *PLoS One* 12:e0183790. 10.1371/journal.pone.0183790 28837643 PMC5570367

[B29] Richards P. J. Connerton P. L. Connerton I. F. (2019). Phage biocontrol of *Campylobacter* jejuni in chickens does not produce collateral effects on the gut microbiota. *Front. Microbiol.* 10:476 10.3389/fmicb.2019.00476 PMC6423408 30930877

[B30] Scott A. E. Timms A. R. Connerton P. L. El-Shibiny A. Connerton I. F. (2007). Bacteriophage influence *Campylobacter jejuni* types populating broiler chickens. *Environ. Microbiol.* 9 2341–2353. 10.1111/j.1462-2920.2007.01351.x 17686030

[B31] Stern N. J. Clavero M. R. Bailey J. S. Cox N. A. Robach M. C. (1995). *Campylobacter* spp. In broilers on the farm and after transport. *Poult. Sci.* 74 937–941. 10.3382/ps.0740937 7644422

[B32] Wagenaar J. A. Van Bergen M. A. Mueller M. A. Wassenaar T. M. Carlton R. M. (2005). Phage therapy reduces *Campylobacter jejuni* colonization in broilers. *Vet. Microbiol.* 109 275–283. 10.1016/j.vetmic.2005.06.002 16024187

[B33] Whyte P. Collins J. D. McGill K. Monahan C. O’Mahony H. (2001). The effect of transportation stress on excretion rates of campylobacters in market-age broilers. *Poult. Sci.* 80 817–820. 10.1093/ps/80.6.817 11441852

