# Supplementary material for: Bacteriophages to Control Campylobacter in Commercially Farmed Broiler Chickens, in Australia
Source: Front Microbiol. 2020 Apr 27;11:632. doi: 10.3389/fmicb.2020.00632 (PMC7197261; doi:10.3389/fmicb.2020.00632)
Supplement: Supplementary file 1 [file Table_1.pdf]

Supplementary Table 1 Sensitivity of pre-screen *Campylobacter* isolates from Farm A and Farm B, to the candidate cocktail bacteriophage

Campylobacter **isolate**	**Candidate bacteriophages**																		
	**PH1**	**PH2**	**PH3**	**PH4**	**PH5**	**PH6**	**PH7**	**PH8**	**PH9**	**PH10**	**PH11**	**PH12**	**PH13**	**PH14**	**PH15**	**PH16**	**PH17**	**PH18**	**P**
**Farm A**																			
1	S	S	S	S	S	S	S	S	I	S	S	I	S	S	S	I	S	S	
2	I	S	S	S	S	I	S	S	I	I	S	I	S	S	S	S	S	S	
3	I	S	S	S	S	I	S	S	I	I	S	I	S	S	S	S	S	S	
4	I	S	S	S	S	I	S	S	I	I	S	I	S	S	S	S	S	S	
5	S	S	S	S	S	I	S	S	I	I	S	S	S	S	S	S	S	S	
6	S	S	S	S	S	I	S	S	I	I	S	I	S	S	S	S	S	S	
7	S	S	S	S	S	I	S	S	I	I	S	I	S	S	S	I	S	S	
8	I	S	S	S	S	I	S	S	I	I	S	I	S	S	S	S	S	S	
9	S	S	S	S	S	S	S	S	I	S	S	I	S	S	S	S	S	S	
10	S	S	S	S	S	S	S	S	I	S	S	I	S	S	S	S	S	S	
**Farm B**																			
1	I	I	I	I	I	I	I	I	I	I	I	I	I	I	I	I	I	S	
2	I	I	I	I	I	I	I	I	I	I	I	I	I	I	I	I	I	S	
3	I	I	I	I	I	I	I	I	I	I	I	I	I	I	I	I	I	S	
4	I	I	I	I	I	I	I	I	I	I	I	I	I	I	I	I	I	S	
5	I	I	I	I	I	I	I	I	I	I	I	I	I	I	I	I	I	S	
6	I	I	I	I	I	I	I	I	I	I	I	I	I	I	I	I	I	S	
7	I	I	I	I	I	I	I	I	I	I	I	I	I	I	I	I	I	S	
8	I	I	I	I	I	I	I	I	I	I	I	I	I	I	I	I	I	S	
9	I	I	I	I	I	I	I	I	I	I	I	I	I	I	I	I	I	S	
10	I	I	I	I	I	I	I	I	I	I	I	I	I	I	I	I	I	S	

S = sensitive to phage; I = insensitive to phage
